# Supplementary material for: Cre-Recombinase Induces Apoptosis and Cell Death in Enterocyte Organoids
Source: Antioxidants (Basel). 2022 Jul 26;11(8):1452. doi: 10.3390/antiox11081452 (PMC9332190; doi:10.3390/antiox11081452)
Supplement: Supplementary file 1 [file antioxidants-11-01452-s001.zip › antioxidants-1789139-supplementary.pdf]

Supplementary Materials

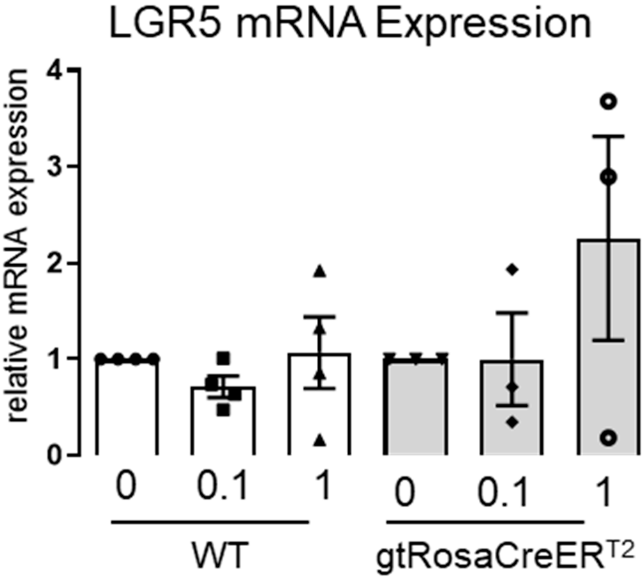

Figure S1. 4-OHT treatment does not affect LGR5 mRNA expression. Quantitative PCR; n = 3–4; mean with SEM; 2-way Anova revealed no significant differences.
